# Inclusion of Antibodies to Cell Culture Media Preserves the Integrity of Genes Encoding RL13 and the Pentameric Complex Components During Fibroblast Passage of Human Cytomegalovirus

**DOI:** 10.3390/v11030221

**Published:** 2019-03-05

**Authors:** Amine Ourahmane, Xiaohong Cui, Li He, Meaghan Catron, Dirk P. Dittmer, Ahmed Al Qaffasaa, Mark R. Schleiss, Laura Hertel, Michael A. McVoy

**Affiliations:** 1 Virginia Commonwealth University, Richmond, VA 23298, USA; ourahmanea@mymail.vcu.edu (A.O.); xiaorobin06@yahoo.com (X.C.); hel3@mymail.vcu.edu (L.H.); catronme@mymail.vcu.edu (M.C.); alqaffasaa@mymail.vcu.edu (A.A.Q.); 2 Department of Microbiology and Immunology, Lineberger Comprehensive Cancer Center Program in Global Oncology, Center for AIDS Research (CfAR), School of Medicine, University of North Carolina at Chapel Hill, Chapel Hill, NC 27599, USA; dirk_dittmer@med.unc.edu; 3 Center for Infectious Diseases and Microbiology Translational Research, Division of Pediatric Infectious Diseases, Minneapolis, MN 55455, USA; schleiss@umn.edu; 4 Children’s Hospital Oakland Research Institute, Oakland, CA 94609, USA

**Keywords:** cytomegalovirus, cell culture, antibodies, adaptation

## Abstract

Propagation of human cytomegalovirus (CMV) in cultured cells results in genetic adaptations that confer improved growth in vitro and significant attenuation in vivo. Mutations in *RL13* arise quickly, while mutations in the *UL128-131A* locus emerge later during fibroblast passage and disrupt formation of a glycoprotein complex that is important for entry into epithelial and endothelial cells. As CMV replicates in the context of host antibodies in vivo, we reasoned that antibodies might mitigate the accumulation of adaptive mutations during cell culture passage. To test this, CMV in infant urine was used to infect replicate fibroblast cultures. One lineage was passaged in the absence of CMV-hyperimmuneglobulin (HIG) while the other was passaged with HIG in the culture medium. The former lost epithelial tropism and acquired mutations disrupting RL13 and UL131A expression, whereas the latter retained epithelial tropism and both gene loci remained intact after 22 passages. Additional mutations resulting in single amino acid changes also occurred in *UL100* encoding glycoprotein M, *UL102* encoding a subunit of the helicase/primase complex, and *UL122* encoding the Immediate Early 2 protein. An epitheliotropic RL13+/UL131A+ virus was isolated by limiting dilution in the presence of HIG and expanded to produce a working stock sufficient to conduct cell tropism experiments. Thus, production of virus stocks by culture in the presence of antibodies may facilitate in vitro experiments using viruses that are genetically more authentic than previously available.

## 1. Introduction

Human cytomegalovirus (CMV) causes a spectrum of diseases in immune-compromised patients, including retinitis in human immunodeficiency virus patients, pneumonitis in transplant patients, and serious birth defects characterized by sensorineural hearing loss and severe mental retardation when acquired during pregnancy. Available options for treating CMV infections are limited, and are hampered by dose-limiting toxicities and the development of resistance. These therapeutic limitations have fostered continued efforts toward the development of effective vaccines, the identification and exploitation of novel targets for antiviral interventions, and the development of passive polyclonal or monoclonal antibody therapeutics.

Since CMV was first isolated in 1957 from clinical samples using cultured human fibroblasts [1], cell culture models of viral propagation and experimentation have made major contributions to our understanding of CMV molecular biology, replication, immunology, and pathogenesis. Many of these contributions were achieved using virus strains that were extensively cell culture-passaged, and are now known to contain substantial genetic deletions, rearrangements, and gene disruptions that were presumably acquired due to selective passage conditions as well as stochastic genetic changes. Recent research has relied increasingly on the use of “clinical-like” strains that lack large deletions or rearrangements, but nevertheless still retain certain changes associated with cell culture propagation. 

Two such changes have been demonstrated to occur consistently during cell culture passage: mutations disrupting the *RL13* open reading frame (ORF) occur irrespective of the cell type used, while mutations in the *UL128-131A* locus emerge during passage in fibroblasts [2]. As the latter disrupt assembly of a complex necessary for infection of epithelial and endothelial cells, they do not generally occur during culture in these cell types [2]. Additional adaptive mutations causing amino acid substitutions or impacting noncoding gene-regulatory regions can also arise, although less consistently [2,3,4].

Given that CMV replication in vivo generally occurs in the context of CMV-specific antibodies, we reasoned that virus propagation in cell culture would more accurately model replication in vivo if CMV-specific antibodies were present in the culture medium. Consequently, the accumulation of certain adaptive mutations might also be mitigated. Here, we report that a CMV clinical isolate serially passaged more than twenty times in fibroblasts cultured in the presence of CMV-hyperimmunoglobulin (HIG) retained epithelial tropism and lacked mutations disrupting *RL13* or genes in the *UL128-131A* locus, but acquired polymorphisms in *UL100* encoding glycoprotein M (gM), *UL102* encoding a protein associated with the helicase/primase complex, and *UL122* encoding the Immediate Early 2 (IE2) protein. A clonal virus retaining the genotypic and phenotypic properties of the parental stock was isolated by limiting dilution and expanded to produce working stocks with titers sufficient to conduct cell tropism experiments in vitro.

## 2. Materials and Methods

### 2.1. Human Subjects and Clinical Sample Collection

CMV culture-positive urine sample-designated KG urine was obtained from a congenitally infected newborn seen at the University of Minnesota Medical Center. KG urine was clarified from cellular debris by centrifugation at 2600× *g* for five minutes, then adjusted to 100 mM sucrose, aliquoted, and stored in liquid nitrogen. Informed consent was obtained from the guardian, and protocols were approved by the Committees for the Conduct of Human Research at Virginia Commonwealth University and University of Minnesota.

### 2.2. Cells

Human MRC-5 fetal lung fibroblasts (ATCC CCL-171) and ARPE-19 retinal pigment epithelium cells (ATCC CRL-2302) were obtained from ATCC and propagated in high-glucose Dulbecco’s modified Eagle medium (Gibco, Gaithersburg, MD, USA) supplemented with 10% fetal calf serum (HyClone Laboratories, San Angelo, TX, USA), 10,000 IU/L penicillin, and ten mg/L streptomycin (Gibco, Gaithersburg, MD, USA ) (medium). N/TERT-1 human epidermal keratinocytes [5], a gift from Iain Morgan, were propagated using Keratinocyte-SFM medium (Invitrogen, #37010-022, Carlsbad, CA, USA) adjusted to 0.3 mM CaCl_2_ and supplemented with human epidermal growth factor and bovine pituitary extract (KSFM). 

### 2.3. Virus

Two T25 flasks of confluent MRC-5 cells were inoculated with equal volumes of KG urine to establish parallel lineages passaged under different culture conditions. One lineage, designated ϕ-KG, was serially passaged using a conventional protocol [6]. The cultures were monitored visually for cytopathic effect (CPE) until large foci were observed. For the first two passages, cells were trypsinized, mixed with 2.5 × 10^5^ trypsinized uninfected cells, and plated again in a T25 flask. For subsequent passages, cells were trypsinized, sonicated on ice in culture medium, and, as the extent of CPE increased, added in progressively decreasing amounts to T25 flasks containing uninfected confluent MRC-5 cells: 5 mL for five passages, 2 mL for two passages, and 1 mL thereafter. Culture times for each passage were approximately one week.

The second lineage, designated Ig-KG, was serially passaged by transferring one-half of the trypsinized infected cells or cell sonicates to T25 flasks containing uninfected confluent MRC-5 cells. One day after each passage, the medium was replaced with the medium containing 2 mg/mL HIG (CytoGam^®^; CSL Behring, King of Prussia, PA, USA). Culture media were not transferred, as they contained neutralizing antibodies and lacked detectable infectious virus. Culture times for each passage were two to three weeks.

Virus stocks were prepared from infected MRC-5 cultures as infected-cell sonicates or culture media supernatants that were clarified by centrifugation at 500× *g* for ten minutes, adjusted to 100 mM sucrose, aliquoted, and stored in liquid nitrogen. Stocks were titrated on MRC-5 cells using a 32-well limiting-dilution method as previously described [7].

Single viruses were isolated from mixed stocks by limiting dilution. Ig-KG passage 22 and ϕ-KG passage 13 stocks were ten-fold serially diluted, and then 96 replicate wells containing confluent MRC-5 cells in 96-well plates were inoculated with 0.1 mL of each dilution. After incubation for one day, the medium in wells inoculated with Ig-KG dilutions was replaced with the medium containing 2 mg/mL HIG, whereas wells inoculated with ϕ-KG dilutions were maintained in the medium lacking HIG. One CPE-positive well, designated ϕ-KG-B5, was selected from the plate that was inoculated with the highest dilution of the ϕ-KG stock that produced CPE-positive wells. ϕ-KG-B5 was expanded using MRC-5 cultures without HIG to produce a culture supernatant stock-designated B5.

One CPE-positive well, designated Ig-KG-H2, was similarly selected from the plate that was inoculated with the highest dilution of the Ig-KG stock that produced CPE-positive wells. Ig-KG-H2 was expanded using MRC-5 cultures containing HIG, as described above. Three stocks of Ig-KG-H2 were produced: (i) stock H2a was prepared by inoculating one T75 flask of MRC-5 cells with Ig-KG-H2, culturing in the presence of 2 mg/mL HIG until extensive CPE was observed, washing the attached cells three times with the medium lacking HIG, then trypsinizing the cells and preparing a clarified infected-cell sonicate as described above; (ii) stock H2b was prepared in the same way from two T75 flasks, but after washing cells were incubated for additional three days in the medium lacking HIG prior to cell harvest and sonicate preparation; (iii) stock H2c was prepared by clarifying the culture medium from the flasks used to make stock H2b.

### 2.4. Cell Infection and Detection of Infected Cells by Immunostaining 

MRC-5, ARPE-19, or N/TERT-1 cell cultures were seeded in 96-well plates. Undifferentiated N/TERT-1 cells were seeded at 2.5 × 10^5^ cells/well and maintained in KSFM for 3–4 days prior to infection. Differentiated N/TERT-1 cultures were prepared as described [8] by seeding N/TERT-1 cells at 2.5 × 10^5^ cells/well in KSFM overnight, incubating cells for 24 h in KSFM with low-concentration CaCl_2_ (0.03 mM), followed by culture for six days with the medium changing every other day in KSFM with high-concentration CaCl_2_ (1.5 mM) but without human epidermal growth factor or bovine pituitary extract. After one hour of incubation with virus stocks, cultures were washed and incubated in the appropriate medium for each cell type. CMV-infected cells were detected by staining for the CMV Immediate Early 1 and 2 (IE1/IE2) proteins using previously described immunofluorescence staining methods [9].

### 2.5. Genetic Sequencing

One T75 flask of confluent MRC-5 cells was inoculated with 0.2 mL ϕ-KG passage 12 stock. After eight days, 11 mL of culture medium from the ϕ-KG-infected flask were transferred to a T225 flask of confluent MRC-5 cells. After nine days of incubation, the culture medium was removed and clarified by centrifugation at 500× *g* for ten minutes. One T75 flask of confluent MRC-5 cells was inoculated with 2 mL of Ig-KG passage 21 stock. After incubation for one day, the medium of the Ig-KG-infected flask was replaced with the medium containing 2 mg/mL HIG. Eleven days after inoculation cells were trypsinized and transferred to a T225 flask containing confluent MRC-5 cells. The next day, the medium was replaced with the medium containing 2 mg/mL HIG. Two weeks after infection, the culture medium was removed and clarified by centrifugation at 500× *g* for ten minutes. Virion-associated DNA was isolated by ultracentrifugation of virions from clarified culture supernatants followed by DNase I treatment, phenol/chloroform extraction, and ethanol precipitation, as described previously [10].

Virion DNA (100 ng) purified from ϕ-KG or Ig-KG cultures was sheared, ligated to barcoded adapters, and size-selected for products in the ~685 bp range, then PCR-amplified for 8–9 cycles and sequenced using an Ion S5 System. The two modal read lengths were 280 bp and 550 bp at >5 million reads per sample. Reads were de novo assembled using Newbler v2.9 and the resulting contigs were mapped to NC_006273/Merlin to yield a draft genome. All reads were re-mapped to the draft genome and a consensus was derived using Geneious v10.1.3 to produce sequences for ϕ-KG passage 13 and Ig-KG passage 22 genomes. The median coverage was >500×. A similar approach was used to generate sequences for ϕ-KG-B5 and Ig-KG-H2 genomes. Differences between ϕ-KG passage 13, Ig-KG passage 22, ϕ-KG-B5, and Ig-KG-H2 identified by whole genome sequencing were confirmed by targeted PCR amplification of affected regions and Sanger sequencing (Eurofins Genomics, Louisville, KY, USA) of both strands. PCR amplification and Sanger sequencing of the KG urine and KG lineage samples were also used to determine the passage number when each mutation likely arose.

### 2.6. Amino Acid Sequence Prediction and Alignments

Sequence analyses were performed with the MacVector 16 sequence analysis software package. *UL100*, *UL102*, and *UL122* exon 5 sequences were obtained from whole genome sequence files of CMV strains Merlin (NC006273), TR (AC146906), JHC (HQ380895), TB40-BAC4 (EF999921), Towne (GQ121041), AD169 (BK000394), UxcA (KX544840), Toledo (AC146905), and FIX (AC146907). IE2 amino acid sequences for each strain were predicted from the exon 5 nucleotide sequences. The F376 polymorphism in JHCp37 [11] was manually created by editing the strain JHC-predicted sequence. The resulting amino acid sequences were aligned using ClustalW. The NCBI database was searched for files containing *UL100*, *UL102*, or *UL122* exon 5 sequences using BLASTn. The retrieved sequences were aligned to the ϕ-KG passage 13 and Ig-KG passage 22 sequences, and then scanned visually for matches.

## 3. Results

### 3.1. HIG in the Culture Medium Prevents Loss of Epithelial Tropism during Fibroblast Passage

Serial fibroblast passage of CMV clinical isolates results in viral mutants that have lost the capacity to infect epithelial and endothelial cells. This phenotypic change arises from mutations that disrupt expression of one of three proteins, UL128, UL130, and UL131A, which combine with glycoproteins H and L (gH and gL) to form a pentameric complex that is necessary for efficient entry into epithelial and endothelial cells, but dispensable for entry into fibroblasts [12,13,14,15,16]. Such mutants emerge around passage 15 and become dominant within a few additional passages [2].

To determine if antibodies can mitigate the selective pressures that promote the emergence of these mutations, a CMV culture-positive urine from a congenitally infected infant was inoculated into replicate flasks of MRC-5 fibroblasts. One lineage (designated ϕ-KG) was serially passaged by a conventional protocol without CMV antibodies added to the culture medium. The other (designated Ig-KG) was also serially passaged, but after each passage, the culture medium was replaced with the medium containing 2 mg/mL of HIG. 

**Figure 1:**
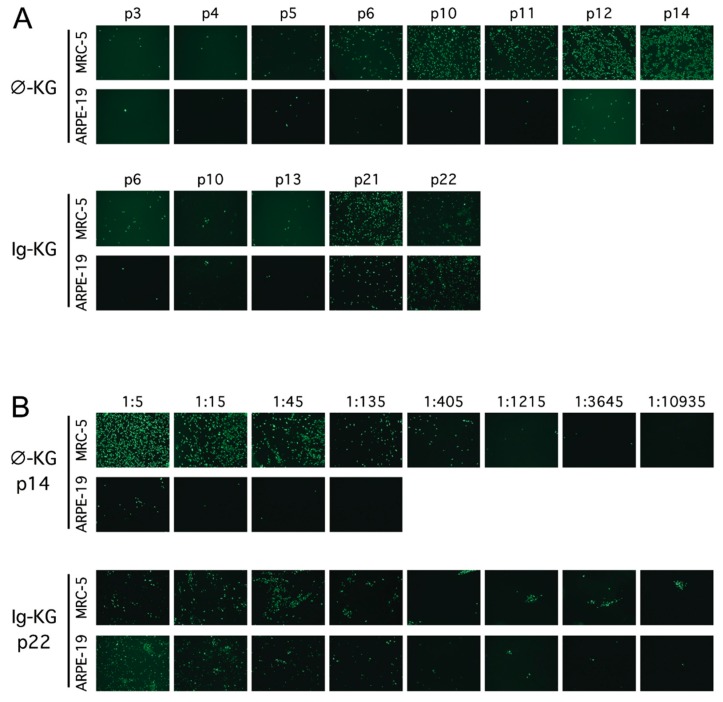
A clinical CMV isolate passaged in the presence of antibodies retains epithelial tropism. (**A**) MRC-5 fibroblasts or ARPE-19 epithelial cells were infected with replicate virus stocks from the indicated passages of ϕ-KG (clarified culture medium) or Ig-KG (infected cell sonicates) and stained for CMV IE1/IE2 after three days. (**B**) The ϕ-KG passage 14 stock and Ig-KG passage 22 stock were serially diluted, added to MRC-5 fibroblasts or ARPE-19 epithelial cells, and stained for CMV IE1/IE2 antigens after three days. Images were taken using a 10× objective.

To assess changes in fibroblast and epithelial tropisms during serial passage of each lineage, matching amounts of stocks prepared from intermediate passages were added to MRC-5 or ARPE-19 cultures. After three days, cultures were stained by immunofluorescence to detect expression of the IE1/IE2 proteins as an indication of successful viral entry and initiation of gene expression. MRC-5 and ARPE-19 cultures inoculated with ϕ-KG passages 3, 4, and 5 exhibited low but similar numbers of IE1/IE2-positive cells, suggesting that the efficiency of viral entry and progression to IE1/IE2 protein expression was similar for the two cell types. However, at ϕ-KG passage 6 and higher, IE1/IE2-positive MRC-5 cells were abundant while IE1/IE2-positive ARPE-19 cells were rare, suggesting a decline in epithelial tropism (Figure 1A, top). In contrast, in cultures inoculated with Ig-KG lineage samples, the numbers of IE1/IE2-positive MRC-5 and ARPE-19 cells remained comparable even up to passage 22, suggesting that viruses in Ig-KG lineage retained tropism for epithelial cells (Figure 1A, bottom). To directly compare fibroblast and epithelial cell infection efficiencies, the ϕ-KG passage 14 and Ig-KG passage 22 stocks were three-fold serially diluted and matching amounts were added to MRC-5 and ARPE-19 cultures. The ϕ-KG passage 14 stock displayed a profound deficiency (approximately 81-fold) in epithelial infection compared to fibroblast infection (Figure 1B, top), while the Ig-KG passage 22 stock infected both cell types with similar efficiencies at each dilution (Figure 1B, bottom).

### 3.2. Genetic Analysis of ϕ-KG and Ig-KG

Loss of epithelial tropism in the ϕ-KG lineage is consistent with literature data reporting that viruses with mutations disrupting expression of pentameric complex components emerge and eventually dominate during fibroblast passage [2]. The fact that the Ig-KG lineage remained epitheliotropic after 22 passages suggests that viruses with pentameric complex mutations fail to emerge during passage in the presence of HIG. To confirm this assumption, DNA was isolated from the supernatants of cells infected with ϕ-KG passage 13 or Ig-KG passage 22 stocks and subjected to Ion Torrent sequencing. ClustalW alignment of the resulting genome sequences revealed differences in nine annotated ORFs. Targeted PCR/Sanger sequencing resolved four of these differences as sequencing errors, while confirming sequence differences in five ORFs: *RL13*, *UL131A*, *UL100*, *UL102*, and *UL122* (Table 1).

*RL13.* The *RL13* ORF in ϕ-KG passage 13 contained a 10 bp deletion, causing a frameshift and consequent truncation of RL13 after amino acid 164. Surprisingly, the Ig-KG passage 22 *RL13* ORF lacked this deletion and was otherwise intact, suggesting that KG virus passaged in the presence of HIG did not acquire mutations disrupting RL13 expression. Targeted PCR/Sanger sequencing of *RL13* in the KG urine and ϕ-KG lineage samples established that the deletion arose between passages 6 and 10 (Table 1). 

*UL131A.* The *UL131A* ORF in ϕ-KG passage 13 contained a single nucleotide insertion frameshifting the amino-acid sequence of the UL131A protein after amino acid 27. In the Ig-KG passage 22 sequence, *UL131A*, as well as ORFs encoding the other two pentamer subunits, UL128 and UL130, were intact and encoded full-length proteins. These results are consistent with the lack of epithelial tropism of viruses in the ϕ-KG passage 13 stock, as the mutation in *UL131A* should prevent expression of UL131A, and hence assembly of a functional pentameric complex. Targeted PCR/Sanger sequencing of *UL131A* sequences in the KG urine and ϕ-KG lineage samples determined that the deletion arose between passages 6 and 10 (Table 1).

**Figure 2:**
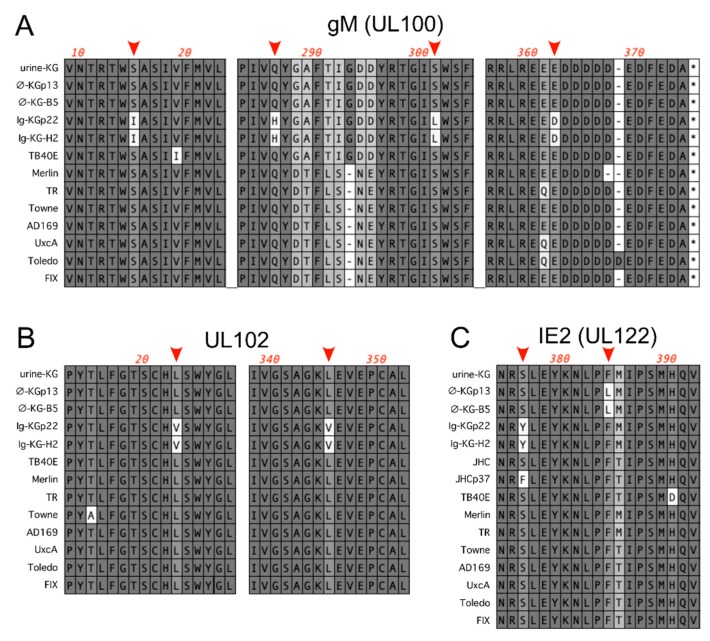
Polymorphisms in gM, UL102, and IE2 arose during serial passage. DNA sequences of the indicated CMV strains were used to predict protein sequences that were then aligned using ClustalW. Selected regions containing polymorphisms of interest (red arrows) are shown for alignments of gM (**A**), UL102 (**B**), and IE2 (**C**).

*UL100*. Four predicted amino acid polymorphisms in gM were detected in the *UL100* ORF of Ig-KG passage 22: S15I, Q286H, S301L, and E362D. By contrast, the ϕ-KG passage 13 *UL100* ORF encoded S15, Q286, S301, and E362. Targeted PCR/Sanger sequencing of KG urine and Ig-KG lineage samples determined that all four mutations arose between passages 13 and 22 (Table 1). S15, Q286, S301, and E362 were 100% conserved among a panel of common CMV strains (Figure 2A) as well as in 41 gM protein sequences identified by a BLAST search of the NCBI database [17].

*UL102*. The *UL102* ORF in Ig-KG passage 22 encoded three nucleotide changes, of which two resulted in predicted amino acid polymorphisms in the UL102 protein, L23V and L345V, while the third, C1812T, was silent. Targeted PCR/Sanger sequencing of KG urine and Ig-KG lineage samples determined that all three mutations arose between passages 13 and 22 (Table 1). L23 and L345 were 100% conserved among a panel of common CMV strains (Figure 2B) and among 100 UL102 sequences identified by a BLAST search of the NCBI database [17].

*UL122*. The *UL122* ORFs in ϕ-KG passage 13 and Ig-KG passage 22 each encoded different single amino acid polymorphisms in the IE2 protein: S376Y in Ig-KG passage 22 and F384L in ϕ-KG passage 13 (Figure 2C). Targeted PCR/Sanger sequencing of KG urine and Ig-KG and ϕ-KG lineage samples determined that in both cases, the mutations arose between passages 6 and 10 of their respective lineages (Table 1). S376 and F384 were 100% conserved among a panel of common CMV strains (Figure 2C) and among all but one of 322 IE2 sequences identified by a BLAST search of the NCBI database [17]. The single exception was an S376F polymorphism that emerged during serial fibroblast passage of strain JHC [11] (Figure 2C).

### 3.3. Viruses Isolated and Expanded from Mixed Stocks

The above analyses used mixed stocks produced after 13 or 22 passages. To improve uniformity, individual viruses were isolated from the mixed stocks by limiting-dilution in 96-well MRC-5 cultures. Virus ϕ-KG-B5 was isolated from the ϕ-KG passage 13 stock and expanded in the absence of HIG. A final working stock was prepared from the clarified cell culture medium. Virus Ig-KG-H2 was similarly isolated and expanded from the Ig-KG passage 22 stock in the presence of HIG. Three working stocks of the Ig-KG-H2 virus were produced and evaluated: (i) stock H2a was prepared as an infected cell lysate immediately after washing to remove HIG; (ii) stock H2b was prepared as an infected cell lysate three days after HIG was removed by washing; (iii) stock H2c was prepared from the clarified cell culture medium from the flasks used to make stock H2b. Titers and other properties of these stocks are shown in Table 2; additional details can be found in Materials and Methods.

Titers of Ig-KG-H2 cell lysate stocks H2a and H2b were low but within the practical range for experimental use (Table 2). Consistent with the presence of neutralizing antibodies, infectious virus was not detected in the culture medium from Ig-KG-H2-infected cultures that contained HIG. However, a low level of infectious Ig-KG-H2 virus (5.0 × 10^2^ pfu/mL) was detected in the H2c culture medium stock produced three days after HIG removal (Table 2).

**Figure 3:**
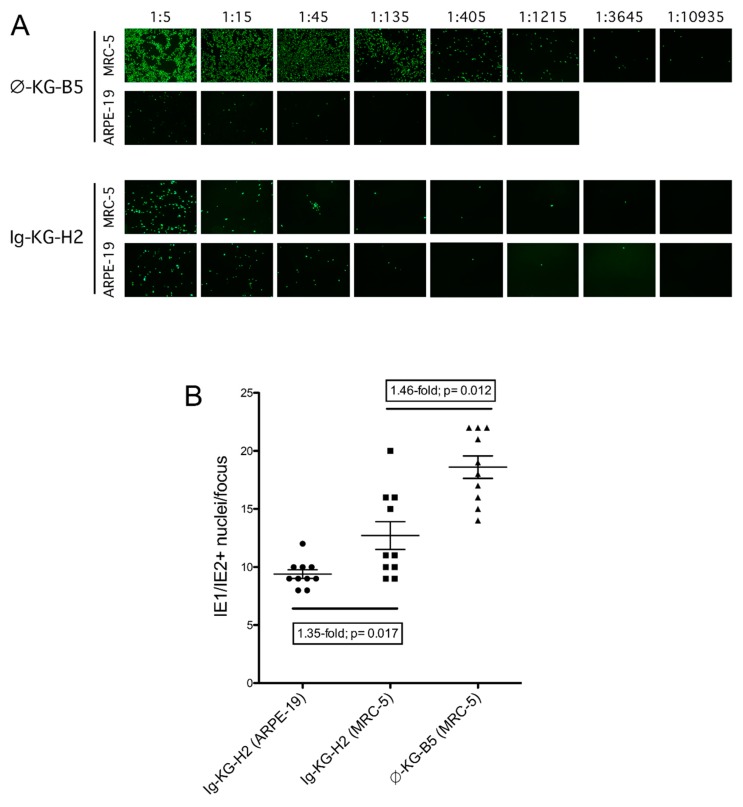
Subcloned viruses retain the tropisms of their parental stocks. (**A**) Viruses ϕ-KG-B5 and Ig-KG-H2 were isolated from ϕ-KG passage 13 or Ig-KG passage 22 stocks, respectively, and then evaluated for infection of MRC-5 or ARPE-19 cells as described in the legend for Figure 1B. Images were taken using a 10× objective. (**B**) The numbers of IE1/IE2-positive nuclei in ten foci from MRC-5 or ARPE-19 cultures infected with ϕ-KG-B5 or Ig-KG-H2 were manually counted. Fold differences in the means and *p*-values (two-tailed *t*-test) are indicated. Stocks B5 and H2a were used in both experiments.

The ϕ-KG-B5 and Ig-KG-H2 viruses recapitulated the epithelial tropisms of their mixed parental stocks. Virus ϕ-KG-B5 infected epithelial cells with very poor efficiency compared to fibroblasts, while Ig-KG-H2 infected both cell types with similar efficiencies (Figure 3A). In addition, Ig-KG-H2 foci appeared to be larger on MRC-5 cells than on ARPE-19 cells, and ϕ-KG-B5 foci appeared larger than those of Ig-KG-H2 on MRC-5 cells [18]. Counting the number of IE1/IE2-positive nuclei per focus revealed that Ig-KG-H2 indeed produced 1.35-fold larger foci on MRC5 cells than on ARPE-19 cells, while ϕ-KG-B5 foci were on average 1.46-fold larger than those of Ig-KG-H2 on MRC-5 cells (Figure 3B), consistent with improved replication efficiencies associated with mutations disrupting expression of RL13 or pentamer components [4,19,20].

DNA sequencing confirmed the presence of frameshift mutations in *RL13* and *UL131A* of ϕ-KG-B5 identical to those identified in the ϕ-KG passage 13 stock, and confirmed the presence of wild type *RL13* and *UL131A* sequences in Ig-KG-H2 identical to those found in KG urine and the Ig-KG passage 22 stock. Similarly, Ig-KG-H2 encoded the same *UL100* and *UL102* mutations that were identified in the Ig-KG passage 22 stock, and Ig-KG-H2 encoded the same S376Y IE2 mutation as the Ig-KG passage 22 stock while ϕ-KG-B5 encoded the same F384L IE2 mutation as the ϕ-KG passage 13 stock.

### 3.4. Experimental Use of CMV Stocks Propagated with HIG

**Figure 4:**
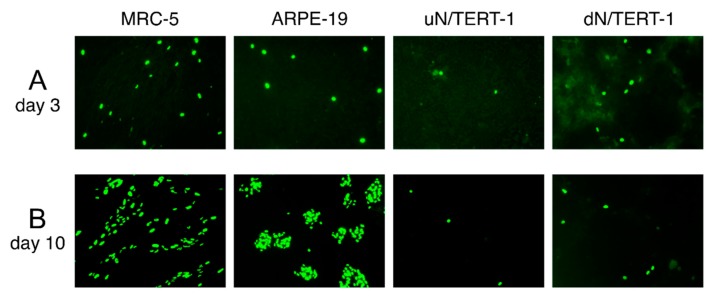
Using Ig-KG-H2 to evaluate CMV tropisms for different cell types. (**A**) 96-well plate cultures of MRC-5, ARPE-19, and N/TERT-1 cells that were either undifferentiated (uN/TERT-1) or differentiated (dN/TERT-1) were inoculated with 500 pfu/well of Ig-KG-H2 (stock H2b) and stained for IE1/IE2 proteins at three days (**A**) or ten days post-infection (**B**). Images were taken using a 10× objective.

To show that stocks generated in the presence of HIG can be used experimentally, cultures of MRC-5, ARPE-19, and undifferentiated or differentiated N/TERT-1 cells were infected with Ig-KG-H2 in order to assess viral tropism for keratinocytes. Infection efficiency, as assessed by counting the number of IE1/IE2-positive cells at day three post-infection, was highest in MRC-5 cells, about two-fold lower in ARPE-19 cells, eight-fold lower in undifferentiated N/TERT-1 cells, and three-fold lower in differentiated N/TERT-1 cells (Figure 4A). Moreover, while the Ig-KG-H2 virus formed foci ten days after infection of MRC-5 cells and ARPE-19 cells, no evidence of focus formation was observed in either differentiated or undifferentiated N/TERT-1 cells (Figure 4B).

## 4. Discussion

For several decades, cell culture-based studies of CMV relied heavily on standard laboratory strains, such as Towne and AD169, which had been adapted through extensive (>100 times) serial passage to replicate efficiently in fibroblasts and to release high levels (10^6^ to 10^7^ pfu/mL) of cell-free virus into the culture medium. In the 1990s, it was discovered that AD169 and a common Towne variant had undergone substantial deletions in the *UL/b’* region: 15.2 kb from AD169 and 13.1 kb from Towne [21,22]. Additional frame-shift mutations disrupting expression of the pentameric complex were later shown to drastically limit replication of these strains in endothelial, epithelial, and certain myeloid lineage cells [12,13,14,15,16,23]. 

More recent studies have trended toward the use of more genetically authentic CMV strains that retain expression of the pentameric complex and/or contain an intact *UL/b’* region. Clinical isolates have also been employed, but these are highly cell-associated during initial passages and produce low titers of cell-free virus (10^3^ to 10^4^ pfu/mL). Replication and release of virus into the culture medium significantly improve with passage [24], making it difficult to predict the properties or degree of authenticity/extent of adaptation of a given stock made at a given passage.

While the mechanism remains unclear, mutations that disrupt RL13 expression appear to contribute significantly toward improved replication and increased release of cell-free virus during cell culture adaptation [2,19,20]. Disruptive *RL13* mutations appear rapidly during passage, and are not specific to one cell type but occur in fibroblast, epithelial, and endothelial cells [2,19]. Mutations that disrupt expression of pentameric complex components also appear to enhance the release of cell-free virus, but primarily arise during serial passage in fibroblasts; viruses passaged in epithelial or endothelial cells generally maintain an intact pentameric complex due to its required role for entry into these cell types [2], although mutations that modulate the levels of pentameric complex expression have been noted [3,4]. Thus, while it is generally possible to preserve expression of pentameric complex components by propagating stocks in ARPE-19 epithelial cells, obtaining stocks of *RL13*+ CMV is challenging, as even low-passage isolates are likely to contain a population of *RL13*-mutant viruses of which prevalence may increase abruptly with passage [19]. An elegant solution was to genetically modify the *RL13* promoter so that, in appropriate host cells, RL13 expression can be conditionally regulated. *RL13*+ stocks can thus be made under repressed conditions and phenotypes associated with RL13 expression can be assessed under induced conditions [19]. 

In the current study, we demonstrate that addition of anti-CMV antibodies to the culture medium allows for the isolation, expansion, serial passage, and sub-cloning of CMV from clinical samples in fibroblasts without accruing mutations disrupting expression of RL13 or assembly of the pentameric complex. While the mechanism by which HIG prevents changes from appearing in the *RL13* and *UL128-UL131A* ORFs during serial passage remains uncertain, it is probable that neutralization of virus released into the culture medium forces virus amplification to occur primarily or even exclusively via cell-to-cell spread, thereby alleviating selective pressures favoring disruption of *RL13* or *UL128-131A*, which promote release of cell-free virions. Analogous effects likely manifest in vivo.

Perhaps not surprisingly, urine-derived virus passaged in the presence HIG did not remain fully wild type. Within the Ig-KG lineage, sequence polymorphisms emerged encoding amino acid substitutions in gM, UL102, and IE2. As viral DNA sequences in KG urine encoded residues at these positions identical to those found in the NCBI database, we surmise that variant genomes emerged within the Ig-KG lineage, either from mutations or through selective expansion of genome variants that were present in KG urine at levels below the limits of detection of the sequencing methods used.

In either case, the selective pressures that favored emergence of these polymorphisms remain unclear. Four gM polymorphisms, S15I, Q286H, S301L, E362D, emerged in the Ig-KG lineage. Within the viral envelope, gM partners with glycoprotein N to form a heterodimer that binds heparin, suggesting a role in mediating viral entry through virion adsorption to cell surface proteoglycans [25,26]. It is therefore tempting to speculate that these amino acid substitutions in gM may somehow promote more efficient cell-to-cell spread of virus within fibroblast monolayers in the context of HIG-containing medium. Although the mechanism is poorly understood, the ability of CMV to spread between cultured fibroblasts in the presence of neutralizing antibodies is well documented [27,28,29,30,31,32,33,34,35]. In contrast, antibodies can inhibit CMV spread within epithelial or endothelial cell monolayers [30,32,33,34,36,37,38,39], and recent work by Murrell et al. demonstrated that viral strains expressing elevated levels of pentameric complex exhibit resistance to antibody inhibition in this setting [20]. Whether gM is involved in this apparent mechanism of immune evasion remains unknown, but could potentially be tested by evaluating the ability of viruses expressing gM sequences from Ig-KG and ϕ-KG to spread in the context of neutralizing antibodies.

It is harder to envision how passage in the presence of HIG might favor the S376Y polymorphism in IE2 or the L23V/L345V polymorphisms in UL102, or why the F384L polymorphism in IE2 arose during passage in the absence of HIG. While an S376F polymorphism in CMV strain JHC was observed to emerge between passages 6 and 37 in fibroblasts (Figure 2C) [11], there appear to be no examples of F384 polymorphisms in the NCBI database. UL102 is a subunit in the helicase/primase complex and as such is involved in DNA replication [40], and while IE2 is known to act as a transactivator of viral early gene transcription [41], it can also bind to the viral origin of replication oriLyt [42] and function in initiation of viral DNA synthesis [43]. Remarkably, a single H390D polymorphism in IE2 renders viral DNA replication independent of UL84, which is essential for DNA replication in viruses with the H390 IE2 allele [44]. This suggests that this region of IE2 may be involved in viral genome replication, and as S376 is near H390 (Figure 2C), it is possible that the polymorphisms in IE2 and UL102 coordinately arose to maintain specific interactions between these two proteins. Thus, for example, the S376Y mutation in IE2 may represent a compensatory mutation in response to polymorphisms in UL102, or vice versa.

Viral stocks produced in the presence of HIG reached moderate titers (~10^4^–10^5^ pfu/mL), but were sufficient for use in experiments that do not require high multiplicities of infection. For example, we were able to use Ig-KG-H2 to evaluate CMV tropism for N/TERT-1 cells (epidermal keratinocytes) and to assess the impact of differentiation on infection efficiency (Figure 4A). Although recent work by Weng et al. showed that a pentameric complex-repaired variant of strain AD169 replicates in undifferentiated oral keratinocytes [45], we did not observe formation of infectious foci in N/TERT-1 cells using either Ig-KG-H2 (Figure 4B) or the same AD169 variant used by Weng et al. [46], suggesting that CMV may have limited ability to spread within epithelial tissues. 

Higher titer stocks would of course be desirable, and may be achievable through further optimization. Additional studies are also needed to determine the minimal HIG concentration required or whether monoclonal antibodies can substitute for HIG. Analogous results might also be achieved, without the use of antibodies, by washing infected cells to remove cell-free virus and passaging only infected cells or cell lysates. 

The culture conditions described here should be useful to extend current CMV genetic systems for evaluation of CMV mutants by allowing for maintenance of an *RL13*+ background. For example, *RL13* mutations could be repaired in existing CMV genomes cloned as bacterial artificial chromosomes (BACs), or new BACs could be derived from *RL13*+ CMV clinical isolates following limited culture in the presence of antibodies. Targeted mutations could then be introduced using *E. coli* genetics and the resulting mutant viruses reconstituted by transfection of fibroblasts in the presence of antibodies. Mutations in non-essential genes could also be directly engineered in RL13+ viruses grown in the presence of HIG using CRISPR/Cas9 without the need for BAC cloning. Studies of this nature should help to elucidate the mechanisms underlying the repressive role(s) played by RL13 during CMV replication in established cell culture systems such as fibroblasts or ARPE-19 cells, and may prove crucial for identifying augmenting or even essential roles for RL13 in other cell types or in vivo. 

## Figures and Tables

**Table 1:** Emergence of mutations during serial passage.

Locus	Lineage	Mutation ^a^	Consequence	Passage Range in Which Mutations Arose ^b^
*RL13*	ϕ-KG	*del* ^c^	frameshift after aa 164	6–10
*UL131A*	ϕ-KG	*ins* ^d^	frameshift after aa 27	6–10
*UL100*	Ig-KG	*G44T*	S15I	13–22
		*G858T*	Q286H	13–22
		*AG901C* *G902T*	S301L	13–22
		*G1086C*	E362D	13–22
*UL102*	Ig-KG	*C67G*	L23V	13–22
		*C1033G*	L345V	13–22
		*C1812T*	silent	13–22
*UL122*	Ig-KG	*C1127A*	S376Y	6–10
	ϕ-KG	*C1152G*	F384L	6–10

^a^ compared to sequences detected in KG urine, ^b^ prevalence of mutations: 0% in lower passage, and 50–100% in higher passage, ^c^ 10 bp deletion, ^d^ 1 bp insertion.

**Table 2:** Virus stocks.

Virus	Stock	Type	Culture Size	Titer (pfu/mL)	Volume (mL)	Total Yield (pfu)
ϕ-KG-B5	B5	medium ^a^	2× T75	6.3 × 10^5^	30	1.89 ×10^7^
Ig-KG-H2	H2a	lysate ^b^	1× T75	6.9 × 10^4^	2	1.38 × 10^5^
	H2b	lysate ^b,c^	2× T75	4.9 × 10^4^	6	2.94 × 10^5^
	H2c	medium ^c^	2× T75	5.0 × 10^2^	30	1.5 × 10^3^

^a^ clarified cell culture medium, ^b^ clarified cell lysates, ^c^ cultures were washed and incubated without HIG for three days before stock preparation.
